# Parasitically coupled 16-port massive MIMO antenna for mmWave applications

**DOI:** 10.1038/s41598-026-55671-x

**Published:** 2026-06-14

**Authors:** Brijesh Mishra, Himanshu Sharma, Neeraj Kumar Misra, Aditya Kumar Singh, T. Y. Satheesha, Sameena Pathan

**Affiliations:** 1 Department of Electronics and Communication Engineering, School of Engineering and Technology, CMR University, Bengaluru, Karnataka India; 2 School of Computer Science & Engineering, IILM University, Greater Noida, 201306 India; 3 School of Electronics Engineering, VIT-AP University, Beside AP Secretariat, Amaravati, 522241, Andhra Pradesh India; 4 Department of Interdisciplinary Courses in Engineering, Chitkara University Institute of Engineering and Technology, Punjab, India; 5 School of Computer Science Engineering, REVA University, Bengaluru, Karnataka 560064 India; 6 Manipal Institute of Technology, Manipal Academy of Higher Education, Manipal, India

**Keywords:** 16-ports, Massive MIMO, mmWave, 56 and 6G, High gain, Spatial diversity and spatial multiplexing, Engineering, Physics

## Abstract

This article presents a 16-port compact (26.2 *×* 79 *×* 0.508 mm$^3$) massive MIMO antenna for mmWave applications. The proposed antenna consists of five triangular-shaped parasitic elements, one triangular-shaped feed-line fed element, and impedance matching network on the radiating plane, whereas defected ground and stubs are used on the ground plane. A systematic study is performed to select an optimal single element antenna (design) among design-1, design-2, design-3 an design-4. Thereafter, a 16-port massive MIMO antenna is implemented using a replica of a single element with modified ground plane structure. The proposed antenna resonates at 40 GHz frequency in mmWave band (37.5–49 GHz) with a 40 dB peak return loss. According to 3GPP bands n259 (39.5–43.5 GHz), n260 (37–40 GHz) and n262 (47.2–48.2 GHz), the resonating band (37.5–49 GHz) of the proposed antenna is part of the upper band of the mmWave spectrum and contains some of the most crucial licensed 5G and beyond wireless communications bands such as 39 GHz, 41 GHz and 47 GHz. Moreover, the proposed antenna achieves a high antenna gain of 17.9 dB and 17.2 dB at 39 GHz and 41 GHz, respectively. In addition, the specific absorption rate analysis of the proposed antenna is conducted by placing the antenna on the right hand of a human male model and 0.045 W/kg of SAR value at 40 GHz is achieved for the proposed antenna. An equivalent circuit of the intended antenna is proposed and simulated using the ADS tool and validated with the results simulated using HFSS.

## Introduction

The unprecedented increase in wireless data traffic through ultra-high-definition video streaming, Internet of Things (IoT) devices, autonomous systems, and immersive technologies including autonomous and virtual reality and similar technologies has necessitated the development of next-generation communication systems^1^. Fifth-generation (5G) and future generation (6G) technologies are likely to provide very high data rates, ultra low latency, enormous connectivity, and spectral efficiency^2^. To meet these strict standards, contemporary communication systems tend to depend more on the mutual benefits of millimeter-wave (mmWave) frequency ranges and Massive Multiple-Input Multiple-Output (Massive MIMO) radio antenna designs^3^. The mmWave communication, which usually also covers between 24 GHz and 100 GHz, has a much broader bandwidth than the conventional sub-6 GHz bands. The greater bandwidth available allows for multi-gigabit per second data rates and mmWave has been shown to be a promising solution to high-capacity wireless connections^4^. Nevertheless, mmWave signals suffer from a number of inherent challenges, including high loss of free-space path, atmospheric absorption, loss caused by obstacle penetration, and minimal diffraction by objects^5^. This implies that the mmWave communication entails highly directional and beam steered transmissions. The massive MIMO system uses a large number of antenna elements at the transmitter and receiver ends to utilize the spatial diversity and spatial multiplexing of the MIMO technique^6^. The large number of antenna elements form a coherent and directional beam in the desired direction, which led to an improvement of the signal-to-noise ratio (SNR) of signal transmission and greatly reduces interference from other directions^7^. These features make it possible to have superior coverage, high data throughput, and effective use of the spectrum. In mmWave systems, massive MIMO is an effective and feasible solution for compact devices due to its small electrical size and ease of installation^8^.

In the literature, limited works have been reported on massive mmWave antennas. several review articles such as tapered slot antenna array fed with a substrate-integrated waveguide (SIW) for mmWave massive system^9^, *8× 8* wideband decoupled mmWave antenna^10^, 32-port super wideband 3D antenna^11^, metamaterial based 16-port non-planar MIMO antenna for sub-6GHz applications^12^, 18-port massive MIMO antenna for smartphone applications^13^, 16-port SIW massive MIMO antenna^14^, 16-port massive MIMO antenna for 6G upper mid-band mobile applications^15^, 16 port indoor base station massive MIMO antenna for sub-6GHz applications^16^, metamaterial-based 16-port massive MIMO antenna for mmWave applications^17^ and 16-port massive MIMO antenna for mmWave application in time division duplex mode^18^. In view of the above study, it has been identified that the massive antenna system, specifically for mmWave applications, is an essential component of next-generation communication technology. However, limited Massive MIMO antennas have been reported in the literature. The works reported in^9^–^18^ present non-planar (3D) and planar antenna geometries for massive MIMO applications; however, these designs suffer from several limitations, including complex structures and control algorithms, atmospheric absorption, loss of free-space path, limited line-of-sight, high loss of insertion and hardware cost, as well as narrow bandwidth, low isolation, and limited gain, which restrict their application in massive MIMO systems into a planar wireless system such as a smartphone^5^.

**Fig. 1:**
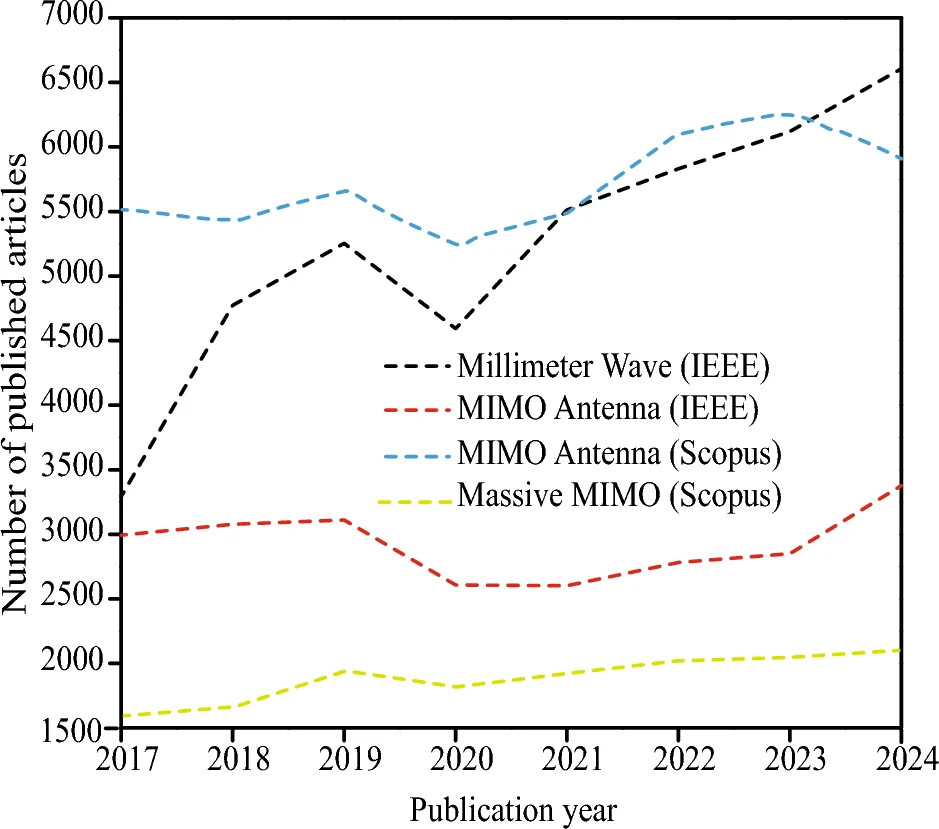
Statistics of mmWave massive MIMO antenna publications in the IEEE and Scopus databases.

Research on mmWave and massive MIMO antennas is a recent trend because these technologies support modern communication devices. A literature report is presented in Fig. 1, which shows statistics on articles published in the IEEE and Scopus databases over the past few years. As shown in Fig. 1, the number of publications in the IEEE and Scopus databases related to mmWave massive MIMO antennas has been growing exponentially. However, the number of published articles decreased in 2020 due to the COVID-19 pandemic and then increased at a rapid pace due to rapid developments in communication technology. In order to fulfill the demand for modern communication devices, the development of an efficient massive MIMO antenna for mmWave applications is a challenging task. A feasible antenna device is a 16-port massive MIMO antenna, which overcomes the limitations of intermediate and large-scale MIMO systems. It comprises sixteen independent antenna ports and therefore can support high spatial multiplexing gain, increased channel capacity, and improved link reliability. A 16-port antenna array can serve multiple users simultaneously using advanced beamforming algorithms, resulting in higher overall system throughput without requiring additional spectrum resources. This is particularly suitable for 5G base stations, small cells, access points, and mmWave backhaul links.

In this article, a 16-port massive MIMO antenna for mmWave applications has been proposed. The proposed antenna is ultra compact (26.2 *×* 79 *×* 0.508 mm$^3$) and unique in design. The proposed antenna offers excellent performance matrices such as wide-band bandwidth of 37.5-49 GHz, high isolation (minimum 20 dB), large antenna gain of 17.9 dB, perfect impedance matching, extremely low ECC value (maximum 0.008), good degree of directive gain (*≈* 10 dB), MEG, TARC and CCL. The whole work is divided into several sections including introduction, development of single port and 16-port massive MIMO antenna, equivalent circuit diagram, s-parameters results, far-field performance, MIMO characteristics, SAR analysis, comparative study and conclusion. In the foregoing sections, each section of this work is discussed in detail with the support of well labeled illustrations. Fabrication and antenna testing could not be performed due to the high cost and unavailability of testing facilities at the upper mmWave band. However, the proposed antenna results have been validated with equivalent circuit results which have been simulated by the ADS tool.

## Development of single port and 16-port massive MIMO antenna design

**Fig. 2:**
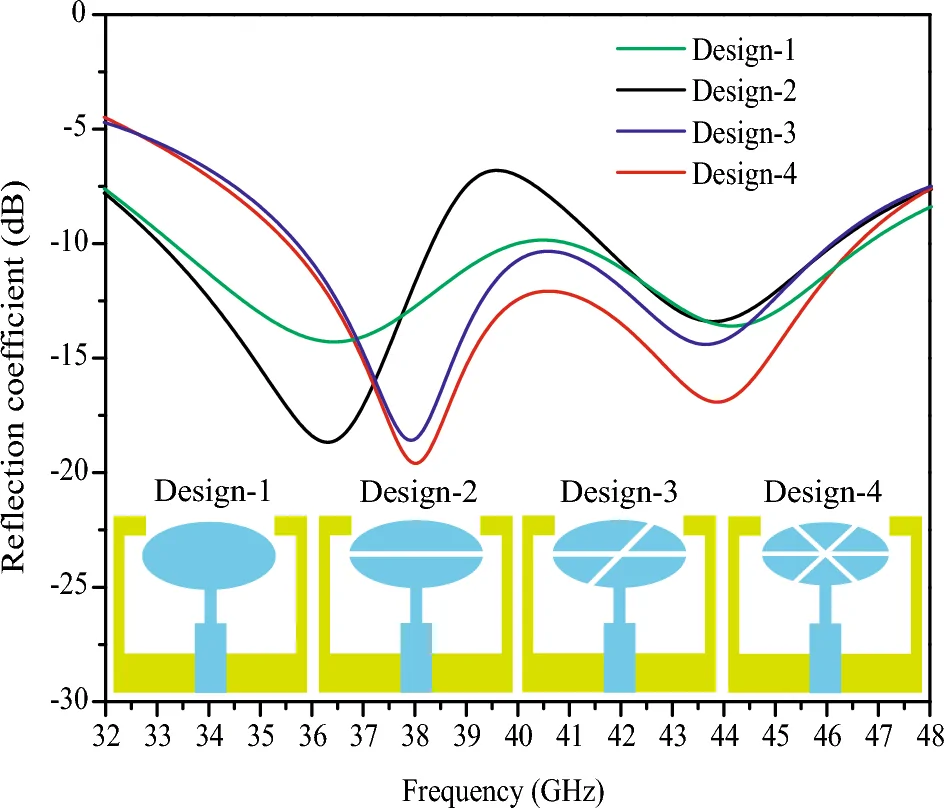
Antenna growth of single port antenna.

The final single element antenna (design-4) is selected from four different designs (design-1, design-2, design-3, design-4) after several systematic studies, clearly illustrated in Fig. 2. The proposed antenna design starts from an antenna geometry in an elliptical shape (design-1) and the physical dimensions of this geometry at 38 GHz are calculated using Eqs. 1, 2, 3, and 4 as reported in the published work^19^.

The antenna is designed over the dielectric substrate of RT/Duroid 5880$^{TM}$ with dielectric constant of 2.33 and height (h) 0.508 mm. 50 *Ω* edge feedline with matching network is used to excite the antenna. In design-1, a horizontal slot (0.25 mm) is introduced to divide the elliptical patch into two equal parts. In this geometry (design-2), the antenna behaves as a fed antenna with parasitic element which reduces the power loss of design-1 but still some portion of the band is greater than -10 dB line. Hence, a diagonal slot (at $\theta _{2}$) is introduced in design-2 named design-3 which improves the performance of design-2 and resonates at 38 GHz and 44 GHz resonating frequencies. Due to the diagonal slot, Design-3 reduces power loss and offers wide bandwidth because diagonal orientation of the slot enables the excitation of multiple resonant modes and supports better polarization characteristics, which collectively enhance radiation efficiency and overall antenna performance.

**Fig. 3:**
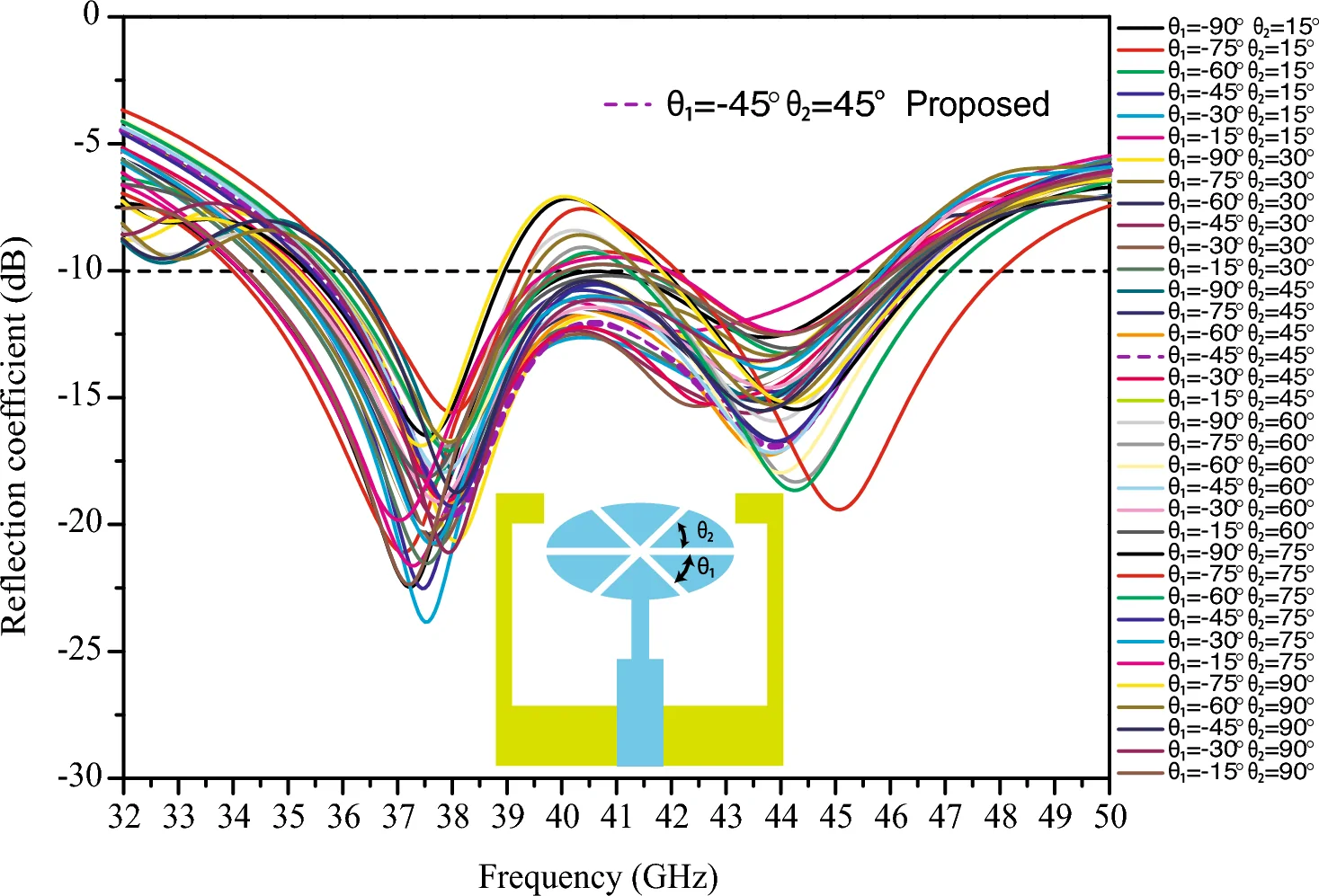
Effect of diagonal slot angles ($\theta _{1}$ & $\theta _{2}$) of design-4 with respect to frequency.

1 $$\begin{aligned} \varepsilon _{\text {eff}} = \varepsilon _r - 0.35\,\varepsilon _r \left( \frac{h}{a} + \frac{h}{b} + \frac{h^2}{ab} \right) \end{aligned}$$

2 $$\begin{aligned} a_{\text {eff}} = \left\{ a^2 + \frac{2ah}{\pi \varepsilon _{\text {eff}}} \left[ \ln \!\left( \frac{a}{2h}\right) + \left( 1.41\varepsilon _{\text {eff}} + 1.77\right) + \frac{h}{a}\left( 0.268\varepsilon _{\text {eff}} + 1.65\right) \right] \right\} ^{1/2} \end{aligned}$$

3 $$\begin{aligned} b_{\text {eff}} = \left\{ b^2 + \frac{2bh}{\pi \varepsilon _{\text {eff}}} \left[ \ln \!\left( \frac{b}{2h}\right) + \left( 1.41\varepsilon _{\text {eff}} + 1.77\right) + \frac{h}{b}\left( 0.268\varepsilon _{\text {eff}} + 1.65\right) \right] \right\} ^{1/2} \end{aligned}$$

4 $$\begin{aligned} f_{m,n} = \frac{c_0 \sqrt{-0.0049e + 3.7888e^{2} - 0.7278e^{3} + 2.2314e^{4}}}{2 \pi a_{eff} \sqrt{\varepsilon _{eff}}} \end{aligned}$$

where, h-substrate height, a-minor axis length, b-major axis length, $a_{eff}$-effective minor axis length, $b_{eff}$-effective major axis length, *\varepsilon*-permitivity, $\varepsilon _{eff}$-effective dielectric constant, $c_{0}$-speed of light.

Furthermore, one more diagonal slot (at $\theta _{1}$) is loaded in design-3 to improve the performance of design-3 further and this modified geometry is named as design-4. Before finalization of design-4, a systematic investigation in terms of reflection coefficient is carried out by varying the angle from $15^{0}$ to $90^{0}$ with a step size of $15^{0}$ for $\theta _{1}$ & $\theta _{2}$. From Fig. 3, it is observed that the antenna design (design-4) with diagonal slot angles $\theta _{1}$ = $\theta _{2}$ = $45^{0}$ offers perfect impedance matching and a low loss reflection coefficient.

### Equivalent circuit diagram of design-4

**Fig. 4:**
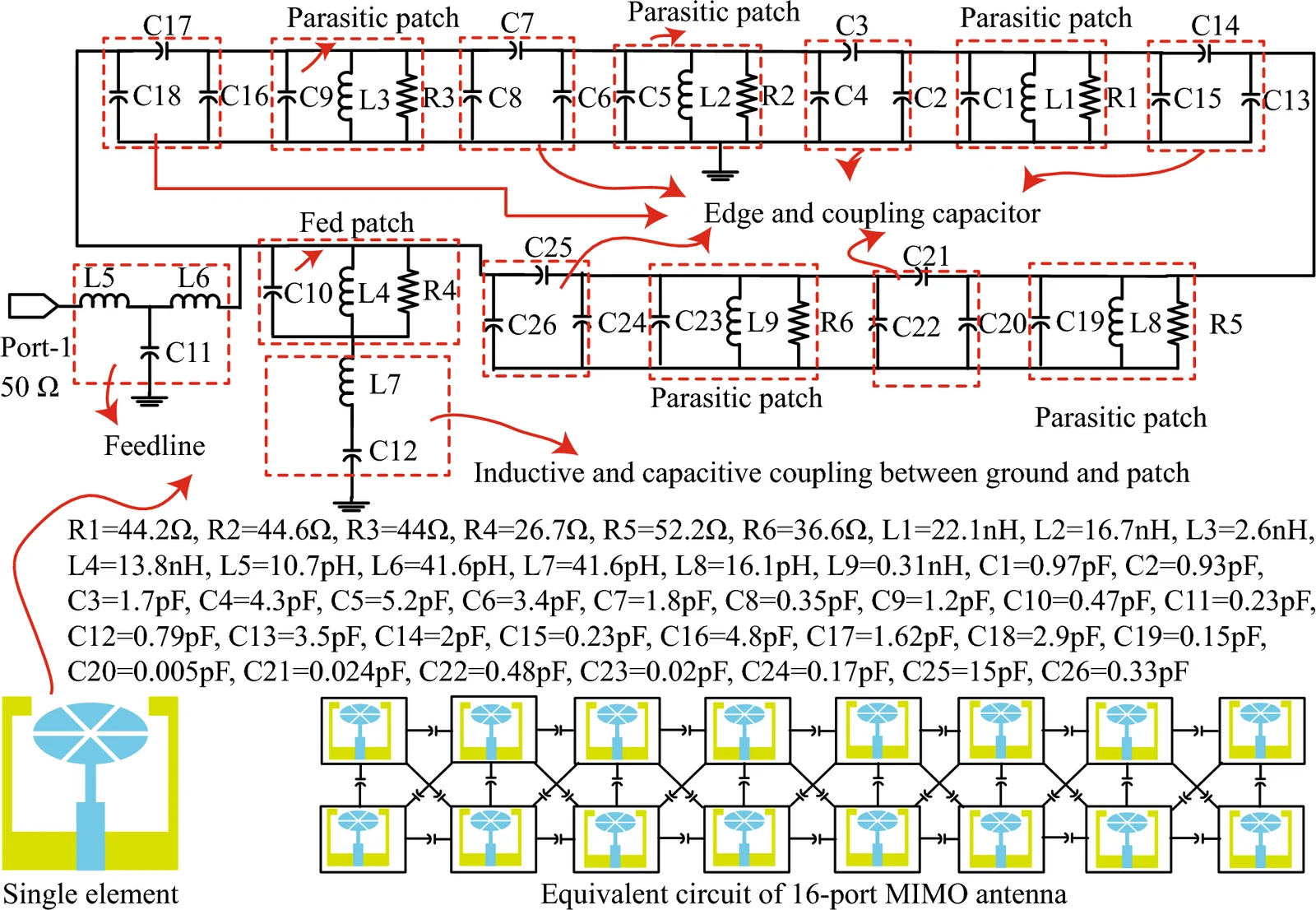
Lumped element equivalent circuit diagram of single element (design-4) and 16-port MIMO antenna.

An equivalent circuit diagram of the proposed single element antenna (design-4) is depicted in Fig. 4. The proposed antenna (Design-4) consists of six radiating elements, wherein five elements are parasitically coupled to one fed element. In the equivalent circuit diagram, the fed and parasitic elements are modeled as parallel resonant RLC tanks, separated by a capacitive *π*-network. The capacitive *π*-network includes edge and coupling capacitors (three capacitors in each section), resulting in a total of 18 capacitive elements. A capacitive network *π* includes edge and coupling capacitors, and these additional capacitances introduce additional resonance modes, which lead to a broadening of the bandwidth^20,21^. In addition, each radiating element consists of one capacitive element in the RLC tank, contributing 6 capacitive elements. A further capacitive element is introduced to represent the capacitive effect between the patch and the ground plane, and another capacitive element is used in the feedline network, which is coupled between two inductors. An equivalent circuit of the proposed single element antenna (design-4) includes five parasitic elements, fed patch, gap between fed and parasitic elements, feedline, ground plane, effect of radiating and ground planes circuits with appropriate arrangement as depicted in Fig. 4. Therefore, the proposed equivalent circuit of the antenna consists of a total of 26 capacitive elements, 9 inductive elements, and 6 resistive elements. The element values of the proposed antenna are optimized using the ADS tool. An optimal value of the circuit elements is listed in Fig. 4.

**Fig. 5:**
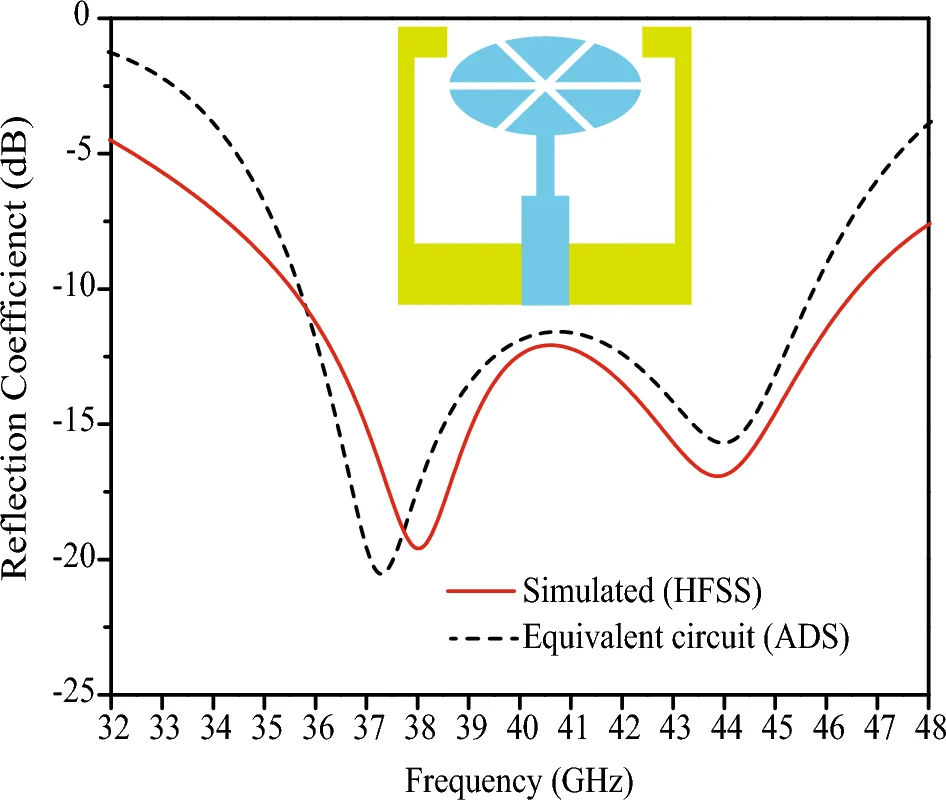
Reflection coefficient performance in terms of HFSS and equivalent circuit of the proposed design.

The equivalent circuit diagram of the proposed antenna design (design-4) is implemented and simulated using the advanced design system (ADS) tool. The reflection coefficient results simulated using HFSS and ADS are presented in Fig. 5. The resonance shift between the HFSS and ADS results arises primarily due to differences in electromagnetic modeling approaches. In the HFSS simulation, the substrate is modeled with full-wave electromagnetic behavior, including its dielectric properties (dielectric constant = 2.33) and loss tangent ($\tan \delta$ = 0.0009), which influences both the effective permittivity and loss characteristics of the antenna. In contrast, the ADS equivalent circuit model is based on a lumped-element approximation, where distributed electromagnetic effects are simplified into ideal or quasi-ideal RLC components. While dielectric losses are partially accounted for through resistive elements in the circuit model, the exact representation of the substrate loss tangent is not explicitly modeled as in HFSS. This simplification can lead to a slight shift in the resonant frequency. Additionally, the ADS model does not fully capture higher-order effects such as fringing fields, surface wave propagation, and complex coupling mechanisms, which are inherently included in HFSS.

A small and acceptable difference between the HFSS and equivalent circuit results is observed in Fig. 5. This deviation in the results is attributed to the different algorithms and assumptions used by ADS and HFSS. The discrepancy between the simulation results of the equivalent circuit implemented in ADS tool and antenna design developed in full-wave electromagnetic simulation tool i.e. HFSS is mainly attributed due to the modeling assumptions and parasitism. The lumped or simplified distributed elements of the antenna design are not a complete set of modes of expression of complex electromagnetic coupling, radiation losses, surface wave interaction, and higher-order modes that exist in a realistic antenna system. In comparison, HFSS conducts a 3D full-wave analysis (finite element method) that is exhaustive, which includes substrate material properties, fringing fields, and geometrical discontinuities accurately. These factors have a more significant effect at mmWave frequencies, where shorter wavelength is more sensitive to the circuit simulator, causing noticeable differences in results between circuit- and full-wave simulation.

### 16-port massive MIMO antenna

**Fig. 6:**
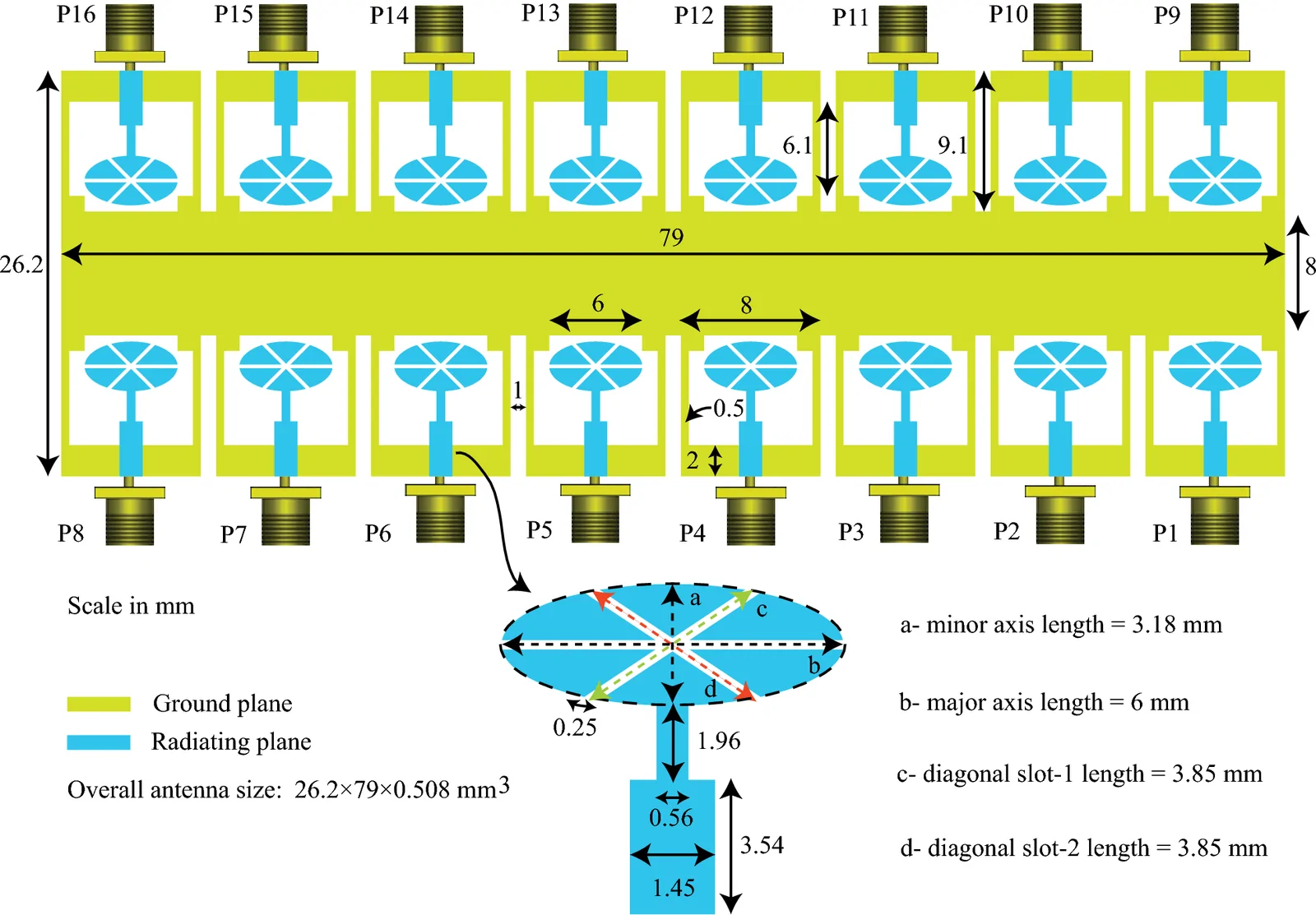
Proposed 16-port massive MIMO antenna geometry.

**Fig. 7:**
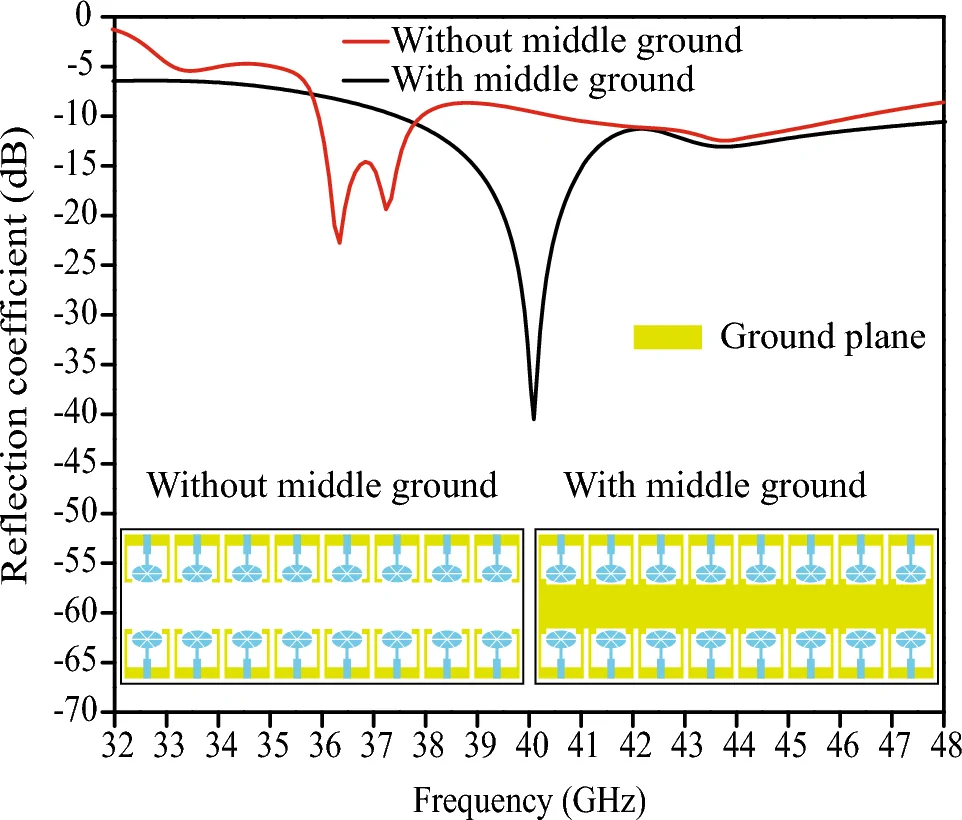
With and without middle ground plane of 16-port massive MIMO antenna.

The proposed single element design is further arranged in a specific manner with 16 elements for a 16-port massive MIMO antenna. In this work, a 16-port massive MIMO antenna configuration is selected for design and study to enhance channel capacity, data rate, and link reliability of mmWave antennas, as these are essential parameters for high-speed 5G/6G wireless communication. At mmWave frequencies, the shorter wavelength allows the integration of a large number of antenna elements into a compact space, which supports increased spatial diversity and enhances beamforming capability. The increased number of ports also facilitates multi-user communication and minimizes the effects of fading and interference, thereby improving spectral efficiency and overall system performance compared to lower-order MIMO configurations. 16 elements of design-4 are placed over a rectangular substrate (26.2 *×* 79 *×* 0.508 mm$^3$) of RT/Duroid 5880$^{TM}$ with dielectric constant = 2.33, loss tangent = 0.0009 and height (h)=0.508 mm. Two adjacent elements are separated with 1 mm gap to produce compact structure and an optimal result for mmWave applications. A rectangular ground plane (8 *×* 79 mm$^2$) between two side elements is connected to make the massive MIMO antenna for real world applications. A well labeled 16-port massive MIMO antenna geometry is presented in Fig. 6. Effect of ground plane of the proposed 16-port massive MIMO antenna is illustrated in Fig. 7. The reflection coefficient performance of the proposed MIMO antenna with and without the middle ground plane structure (8 *×* 79 mm$^2$) is studied. It is observed that the middle ground plane connects all element ground planes and forms a common ground, which is essential for the practical implementation of MIMO systems. Without the middle ground plane, the antenna suffers from multiple harmonics in the reflection coefficient curve, whereas with the middle ground plane, the MIMO antenna system suppresses multiple harmonics and provides smooth resonance peaks at 40 GHz. The 16-port MIMO antenna with a middle ground plane offers improved performance because a split ground plane increases the number of resonant paths and electromagnetic interactions, producing multiple harmonics, whereas a connected ground plane provides a stable current return path that suppresses parasitic modes and neutralizes harmonic resonances.

## Results and discussion

**Fig. 8:**
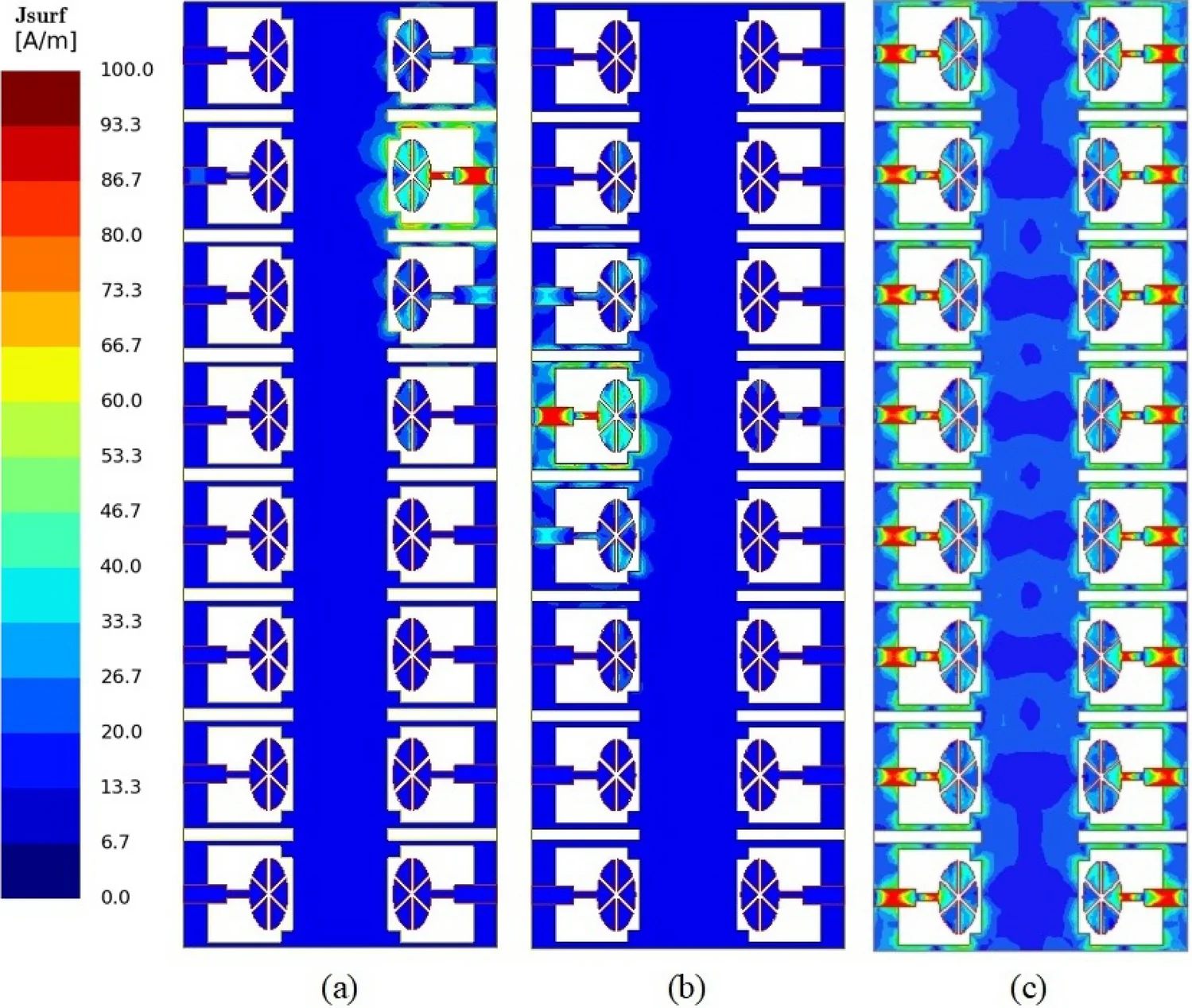
surface current distributions of the intended 16-port antenna at 40 GHz (**a**) if port-2 is excited (**b**) if port-12 is excited (**c**) if all ports are excited.

The surface current distributions of the intended 16-port antenna at 40 GHz is analyzed. The surface current distributions if port-2 is excited only, if port-12 is excited only and all ports are excited simultaneously are depicted in Fig. 8a, b & c, respectively. Figure 8a, b clearly show that each element of the antenna system functions independently as interference between adjacent elements is negligible due to good near-field and mutual couplings. Moreover, all ports are excited simultaneously in Fig 8c, where surface currents are distributed uniformly across all radiating elements, leading to strong electromagnetic coupling and radiating efficiency. The surface current distributions of the proposed antenna show controlled excitation for all ports to radiate a highly isolated field, resulting in high isolation performance of the antenna. As seen, surface current distribution of the proposed 16-port massive MIMO antenna at 40 GHz indicates good excitation of the radiating elements near the feed lines, radiating patches, parasitic edges and ground plane strips with strong concentration of current. The optimized middle ground plane and DGS structures are effectively implemented to suppresses the propagation of undesired surface waves, and thus reduce the electromagnetic interference of adjacent elements.

**Fig. 9:**
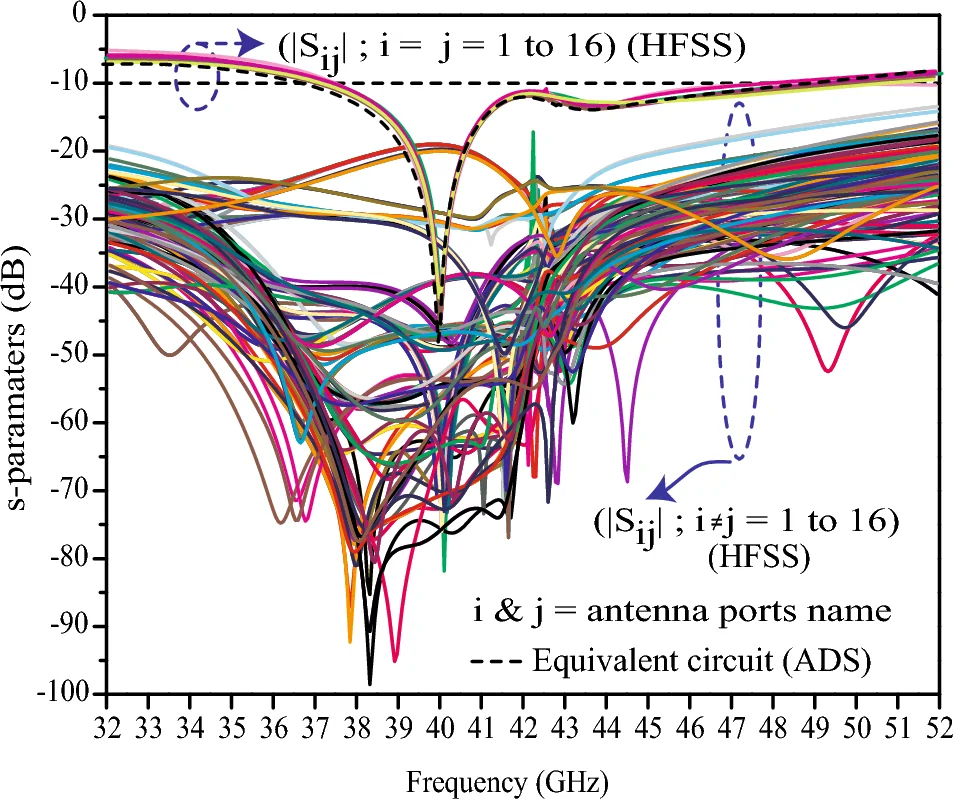
Transmission and reflection coefficient performance of the proposed antenna.

The reflection and transmission coefficient curves of the proposed antenna for all ports are illustrated in Fig. 9. The proposed antenna resonates at 40 GHz frequency in mmWave band (37.5-49 GHz) with a 40 dB peak return loss. According to 3GPP bands n259 (39.5–43.5 GHz), n260 (37-40 GHz) and n262 (47.2-48.2 GHz), the resonating band (37.5-49 GHz) of the proposed antenna is part of the upper band of the mmWave spectrum and contains some of the most crucial licensed 5G and beyond wireless communications bands such as 39 GHz, 41 GHz and 47 GHz^22^. The significance of these bands is that they allow much broader bandwidth than sub-6 GHz bands which enables very high data rate and low latency. Moreover, devices works at this band offer integration of large numbers of devices to connect with each other, which are needed by higher-level applications such as high-capacity mobile broadband, vehicular communications, and next-generation wireless networks. These bands also enable highly directional beamforming and massive MIMO technologies that aid in countering the propagation losses and enhancing spectral efficiency. The transmission coefficient of the proposed antenna is also shown in Fig. 9. In 16-port antenna system, total possible transmission coefficient arrangement is 240 and showing 240 curves in a single plot is a tedious task. Hence, possible number of arrangements of transmission coefficient curves is clearly illustrated in Fig. 9.

The results of the transmission coefficient indicate that the proposed antenna elements are highly isolated from each other because more than 20 dB isolation has been achieved throughout the band. More than 20 dB isolation of the proposed antenna has been achieved due to several factors, such as the selection of an optimal spatial distance (4 mm) between the two elements and the efficient design of the ground plane structure, which behaves as a decoupler and reduces mutual coupling to minimize unwanted energy transfer. The connected ground plane structure in a MIMO system provides a common reference signal for the entire system, as currents excited by one element travel across the ground plane, making it suitable for practical applications^23^. The introduction of a middle ground plane acts as a decoupling structure that controls surface waves and suppresses higher-order mode resonances^24^-^25^. The separation between the two elements is a key parameter for enhancing isolation in a MIMO system; however, increasing this distance may lead to a larger overall antenna size. In this work, an optimal distance of 4 mm between the two elements is chosen to establish a trade-off between performance parameters. The improvement in isolation in the proposed antenna is therefore a combined effect of the connected ground plane, the middle ground structure, and the spacing between the two elements, rather than the spacing alone.

**Fig. 10:**
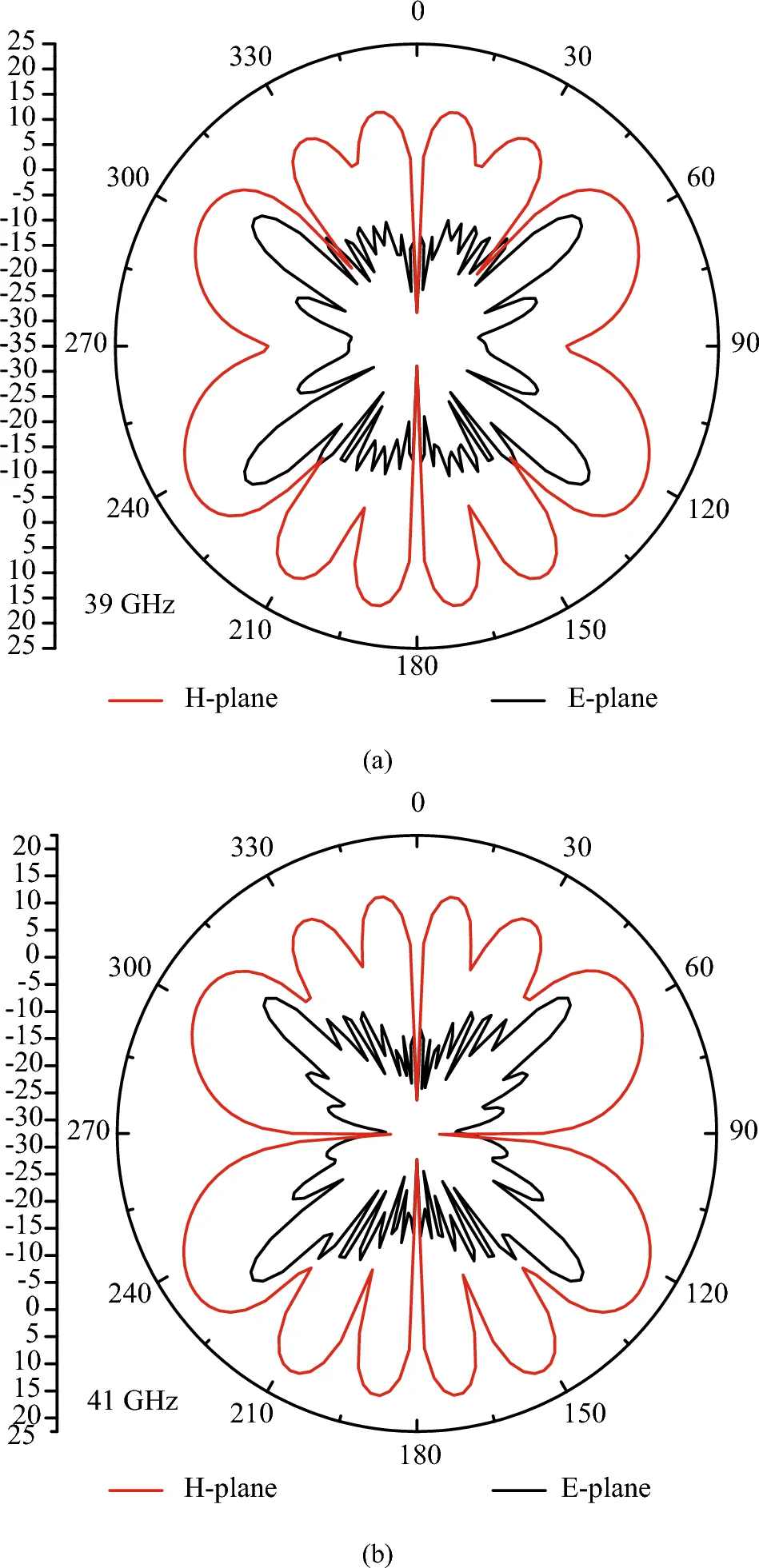
E & H plane radiation patterns at (**a**) 39 GHz (**b**) 41 GHz.

**Fig. 11:**
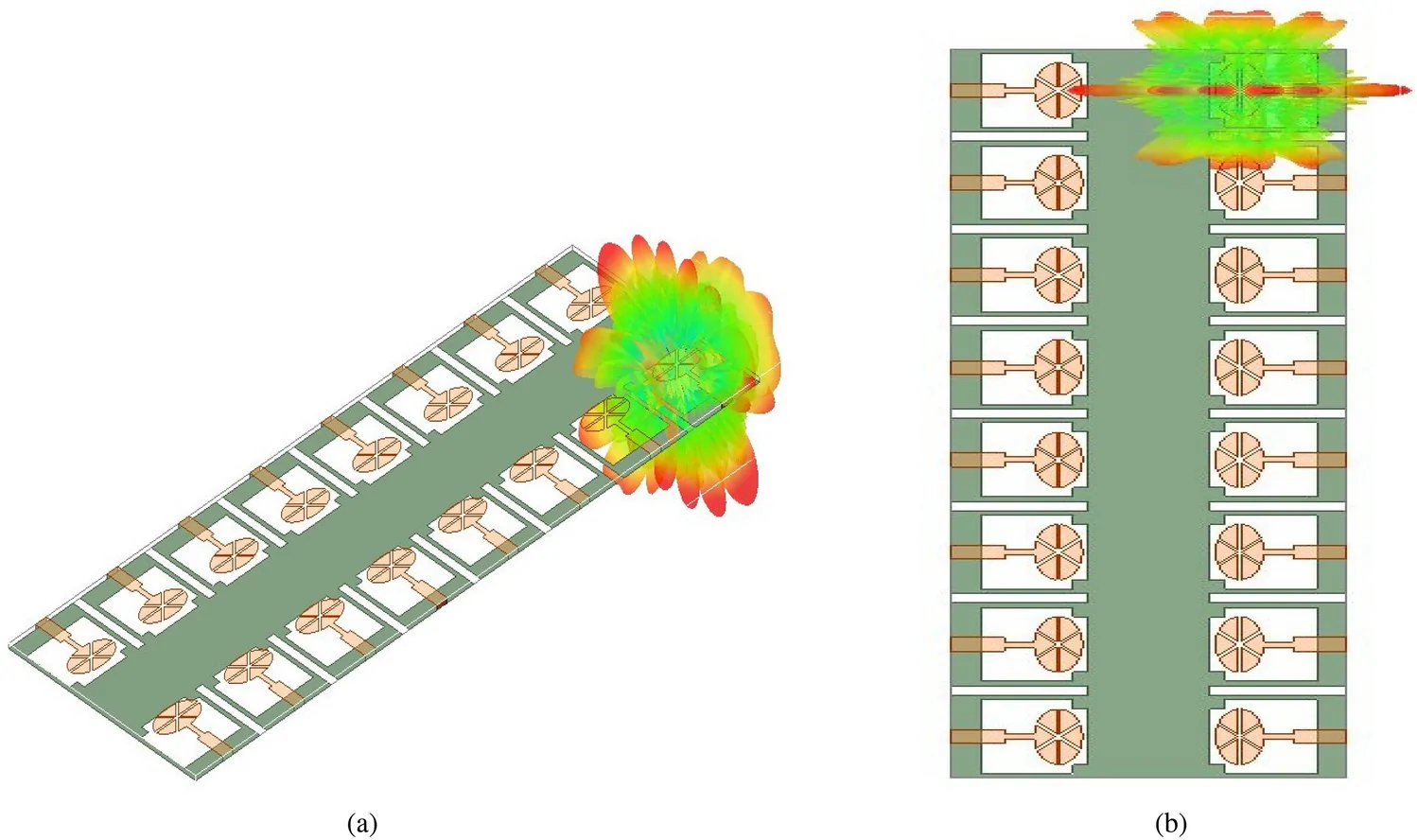
3D radiation pattern at 40 GHz (**a**) isometric view (**b**) front view.

The radiation patterns at two useful frequencies 39 GHz & 41 GHz in terms of E & H plane of the suggested antenna are illustrated in Fig. 10a, b, respectively. The radiation patterns of the E-plane and H-plane are used to demonstrate the directional radiation properties in the two orthogonal planes of the proposed antenna. The E-plane radiation pattern is the change of radiated power in the plane on which the electric field vector and the direction of the maximum radiation are located. These radiations normally represent the main beam shape and beam width of the antenna. The H-plane radiation pattern is the distribution of the radiations in a plane normal to the magnetic field vector, which gives the data regarding the azimuthal coverage of the antenna and the side lobe characteristics. In the case of mmWave antennas, these patterns play a crucial role in evaluating several characteristics such as directivity of the beams, stability of the gain and applicability in high-data rate and beam-steering of communication systems. The proposed antenna shows a stable and bidirectional 8-shape radiation patterns. The advantages of 8-shaped (figure-eight) radiation pattern include bi-directional radiations which means powerful and almost equal radiation in two opposite directions with lower radiation in other directions. This type of patterns are beneficial in the following terms; enhancing coverage, less interference with undesired directions and useful in applications that demand directional communication and polarization diversity. Subsequently, front and isometric view of 3D total radiation pattern at 40 GHz is portrayed in Fig. 11a, b, respectively. Results indicate that the Figs. 10 and  11 are in conformity. Moreover, Figs. 10 and  11 indicate that the proposed antenna exhibits a significantly low level of cross polarization component, which is highly desirable for efficient EM wave transmission.

**Fig. 12:**
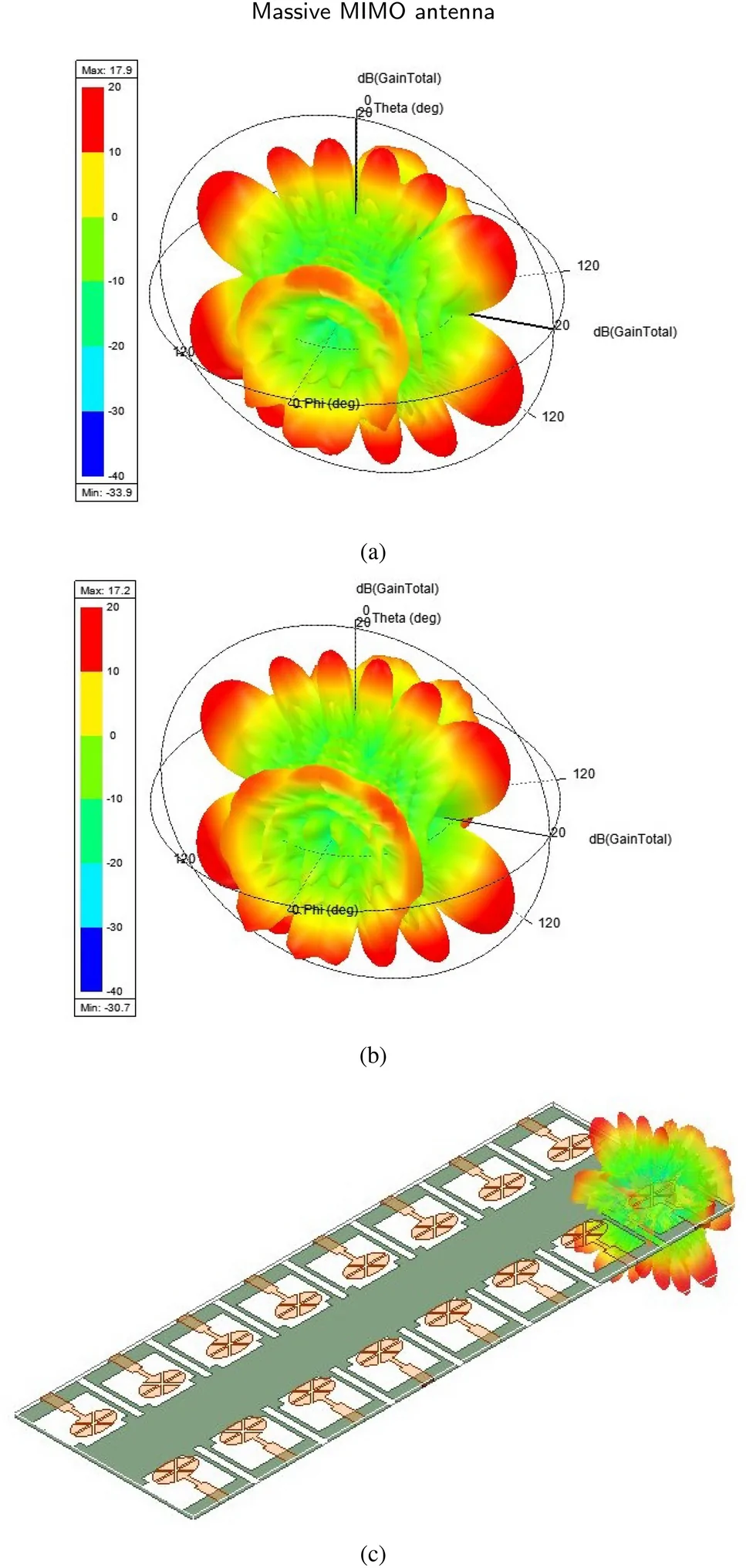
3D gain of the proposed antenna at (**a**) 39 GHz (**b**) 41 GHz (**c**) 3D gain at 40 GHz along with antenna geometry.

A 3D gain of the proposed antenna at 39 GHz and 41 GHz is illustrated in Fig. 12a, b, respectively. The 3D gain of an antenna represents the spatial distribution of the radiated electromagnetic power with respect to an isotropic radiator. According to electromagnetic wave theory, the gain of an antenna depends on both effective aperture and radiation efficiency, which is determined by the propagation of waves, the surface currents, and the distribution of the phase over the antenna circuitry. The shorter wavelength (at mmWave frequency) enables highly directive radiation with narrow beams to achieve higher spatial resolution and spectral efficiency as well as increased link reliability in wireless communication systems operating at high frequencies. The intended antenna exhibits a gain of 17.9 dB and 17.2 dB at the 39 GHz and 41 GHz resonating frequencies, respectively. Moreover, the 3D gain of the antenna as depicted in Fig. 12a,b also agrees with the results presented in Fig. 10a, b. In addition, the gain variation of the proposed antenna along with of the antenna design is shown in Fig. 12c, which is in consonance with Fig. 12a,b.

**Fig. 13:**
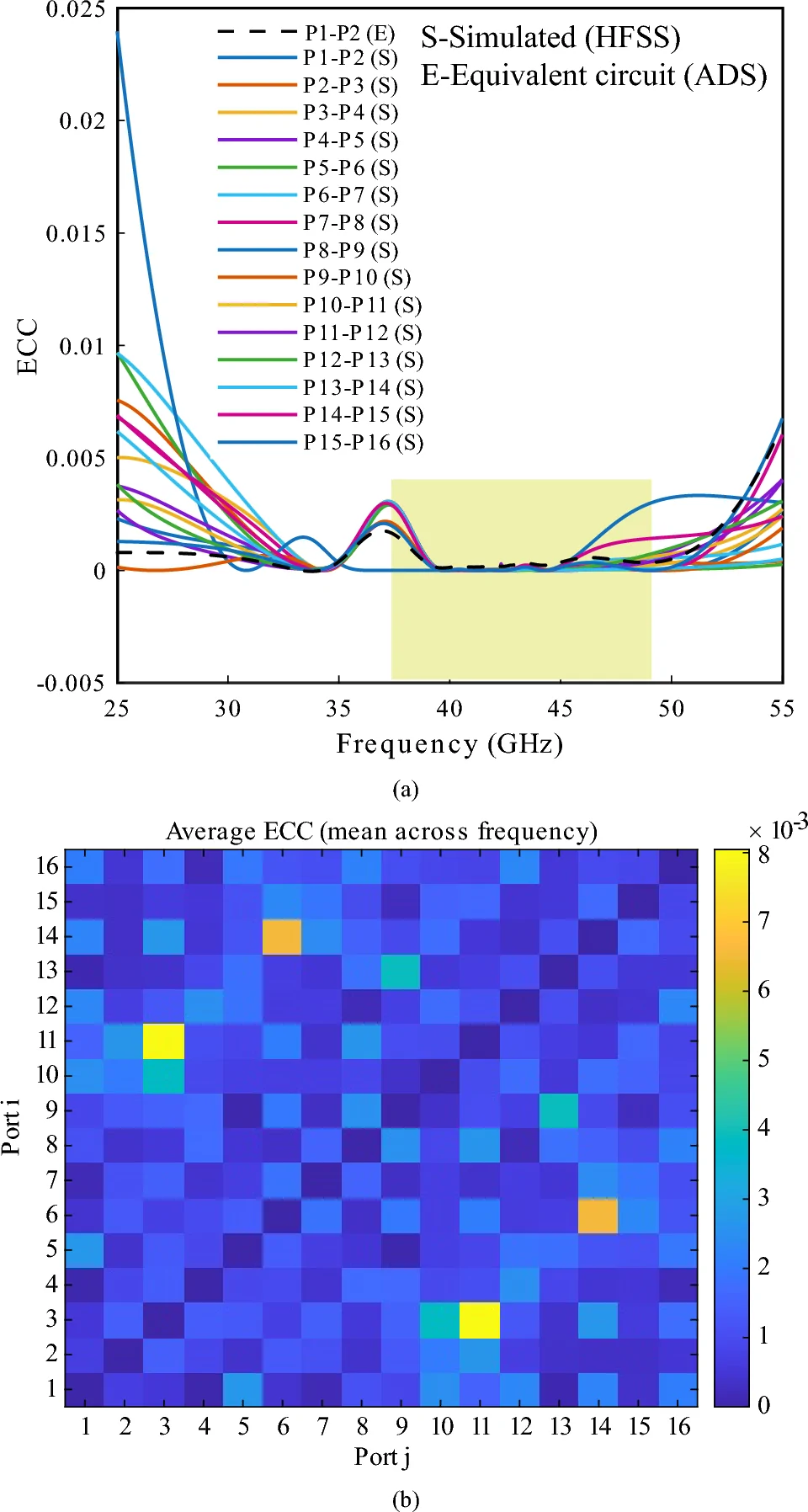
(**a**) ECC for adjacent port pairs (P1-P2 ... P15-P16) (**b**) average ECC heatmap.

### Massive MIMO characteristics

Massive MIMO performance of the proposed antenna including ECC, DG, MEG, TARC, CCL and CC is presented in this section. According to Eq. 5 as extended from the work reported in^26^, the ECC value of the suggested antenna is calculated from the scattering parameters. The adjacent port pairs of the ECC curves and the average ECC heatmap are shown in Fig. 13a, b, respectively. For 16-port massive MIMO antenna, total 240 transmission coefficient arrangements are possible and all port combination is challenging to show in one illustration. Hence, adjacent ports combination (P1-P2 ... P15-P16) is only considered in this study and remaining ports have been omitted from the discussion. However, the average ECC heatmap plot as depicted in Fig. 13b shows a graphical representation of the correlation of all port pairs (256) of 16-port massive MIMO antenna. Corresponding to each pair, the ECC value is indicated on the vertical scale on the right-side of Fig. 13b.

5 $$\begin{aligned} \textrm{ECC}_{i, j, 16} = \frac{\left| \sum _{k=1}^{16} S_{ki}^{*} S_{kj}\right| ^{2}}{\left( 1-\sum _{k=1}^{16}\left| S_{ki}\right| ^{2}\right) \left( 1-\sum _{k=1}^{16}\left| S_{kj}\right| ^{2}\right) } \end{aligned}$$

The ECC value of a 16-port MIMO antenna measures how the signals of two radiating elements are correlated with each other. The MIMO system indicates independent transmission of the signal from multiple elements when its value is zero, while transmission of identical signals in the same direction by multiple elements results in a highly correlated ECC value, i.e. ECC = 1. In the case of adjacent ports near proximity, the ECC will be mainly dependent on near-field electromagnetic coupling, surface current interaction, and overlapping radiation modes, whose occurrence is determined by the physical proximity of elements. The proposed antenna offers a good degree of ECC value which is less than 0.003 and 0.008 for adjacent port pairs and all port pairs. The ECC scale of heatmap is $10^{-3}$ which represents a small value of ECC. However, relatively higher ECC between non-adjacent ports (e.g., Port 3 and Port 11 and Port 6 and Port 14) is observed which is mainly due to the electromagnetic symmetry and similar radiation characteristics of these elements rather than their physical separation. Although they are spatially distant, these ports are positioned in a quasi-symmetric manner within the antenna layout, leading to comparable current distributions and overlapping radiation patterns, which increases correlation. In contrast, several adjacent elements are intentionally arranged with pattern or polarization diversity and appropriate decoupling mechanisms, resulting in lower ECC despite closer spacing. Additionally, surface wave propagation and common ground plane coupling can create indirect interaction paths between electrically similar but physically separated elements. According to work reported in the literature^26^ and^27^, a value of less than 0.5 ECC is considered acceptable limit for efficient transmission. A very low ECC value of the proposed antenna indicates that each element of the antenna system is highly independent of the EM waves to radiate in free space.

**Fig. 14:**
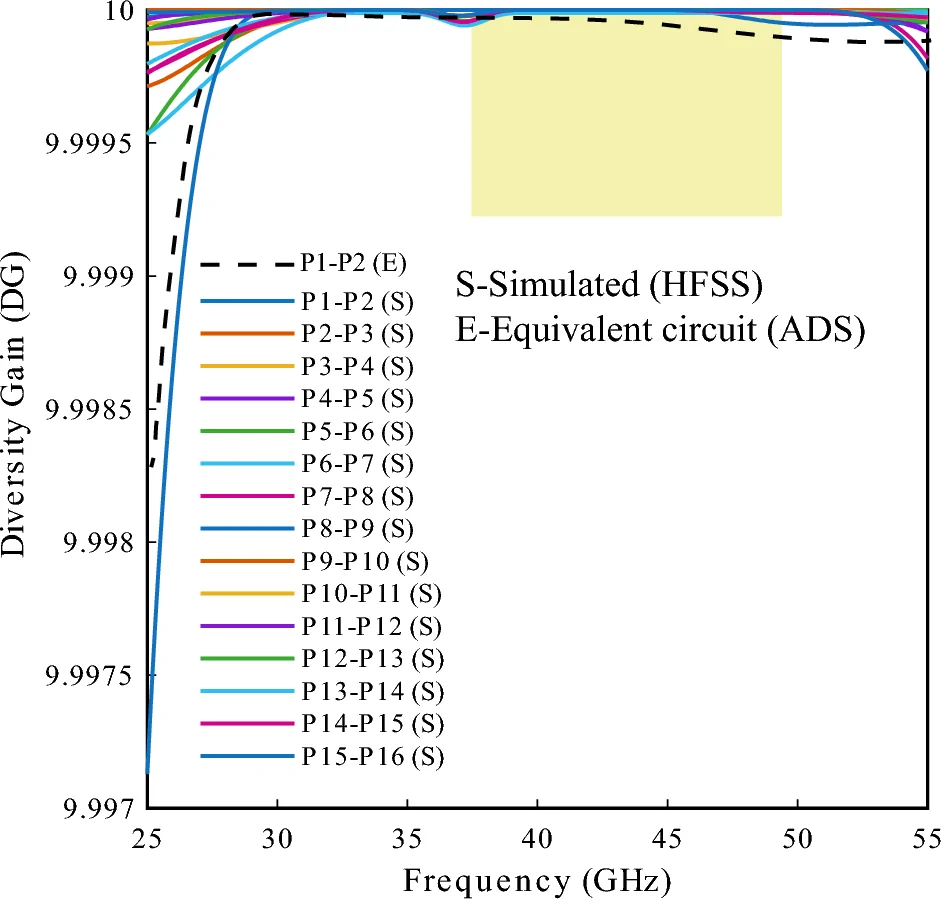
Diversity gain plot between adjacent port pairs.

The diversity gain parameter of the MIMO antenna represents a reliable and quality signal when independent elements transmit or receive signals from multiple paths. In MIMO system, antenna suffers from fading, shadowing, and interference in multipath propagation event affect is more at mmWave frequency^28^. The DG of the proposed antenna is computed from the s-parameters according to Eq. 6 and illustrated in Fig. 14. From Eq. 6 it can be deduced that a low value of ECC gives a good degree of DG value and approaches the ideal value (10 dB) if the value of ECC is zero. The proposed 16-port MIMO antenna attains an approximately ideal DG value due to its low value of ECC. The DG value (*≈* = 10 dB) of the proposed antenna indicates that reliable and quality communication is possible through the proposed 16-port massive MIMO antenna system.

6 $$\begin{aligned} \mathrm {DG\ (dB)}_{i,j,16} = 10 \sqrt{1 - \left| \textrm{ECC}_{i,j,16}\right| ^{2}} \end{aligned}$$

**Fig. 15:**
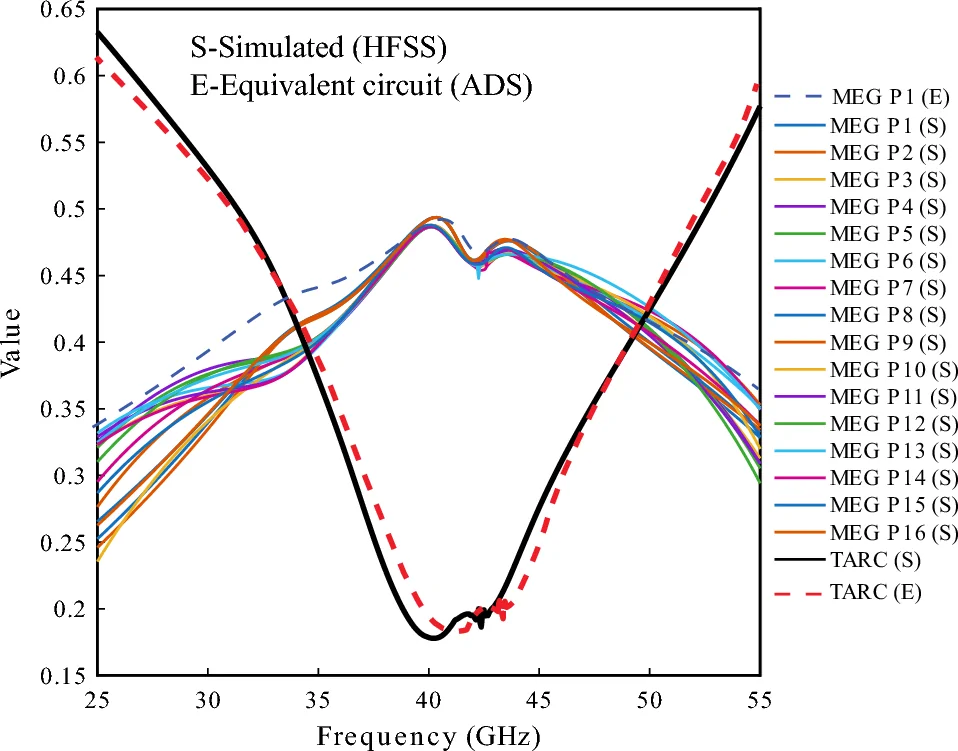
TARC and MEG curves of the suggested antenna system.

**Fig. 16:**
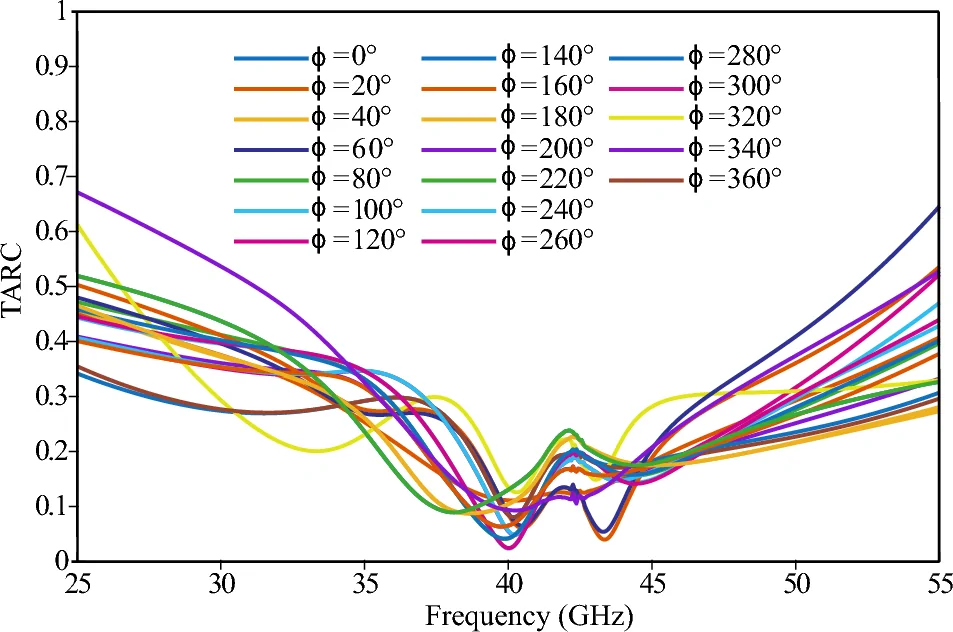
TARC versus frequency plot with phase variation.

The TARC of the proposed antenna is illustrated in Fig. 15 wherein, at *ϕ* = $0^{0}$ the TARC value is chosen and the remaining value of *ϕ* is shaved off the plot to avoid clustering and repetition of the curves and TARC variation with respect to phase (*ϕ*) is illustrated in a separate Fig. 16 for more clarity. The TARC value is computed from s-parameters based on the Eq. 7 which is an extended version of the works reported in^29^ &^30^. The actual prediction of the antenna bandwidth can be done through the TARC curve instead of the reflection coefficient curve because TARC evaluates the combined effect of all ports at specific phase difference (*ϕ*) while the return loss estimates each port individually. Figure 15 confers that the TARC curve is in agreement with the reflection coefficient curve (cf. Fig. 9) because the -10 dB bandwidth of the reflection coefficient and TARC (-10 dB equivalent to 0.316 on linear scale) curves are more or less same. The TARC value (< -10 dB or < 0.316) of the proposed antenna in the operating band is in an acceptable limit^29^. In addition, the TARC value of the proposed antenna is studied for different phase values in the interval of $0^{\circ }$–$360^{\circ }$ with a $20^{\circ }$ step size. The TARC plot of the proposed antenna for different phase values is illustrated in Fig. 16. Phase variation affects the TARC parameter of the proposed antenna when all ports are excited simultaneously; however, this deviation is minimal and within acceptable limits, which confirms the stability of the TARC parameter and the effective operating bandwidth. Moreover, the MEG plot of the intended antenna is also presented in Fig. 15. The MEG curve for each port is computed from the s-parameters as given in Eq. 8 and illustrated in Fig. 15. The MEG of the antenna measures the average power gained with respect to the total available power in free space. In subsequent to this, if MEG of all the ports overlaps to each other, it means that each antenna contributes equally in signal transmission and reception. The results of MEG as shown in Fig. 15 satisfy both the criteria i.e. MEG< -3 dB or 0.707 and overlap each port MEG^29^.

7 $$\begin{aligned} \textrm{TARC} = \frac{\sqrt{\left| \sum _{i=1}^{16} S_{i1} + \sum _{m=2}^{16} S_{im} e^{j\phi _{m-1}} \right| }}{\sqrt{16}} \end{aligned}$$

8 $$\begin{aligned} \textrm{MEG}_{P_i (i=1,2...16)} = 0.5 \left[ 1 - \sum _{j=1}^{16} \left| S_{ij} \right| ^2 \right] \end{aligned}$$

**Fig. 17:**
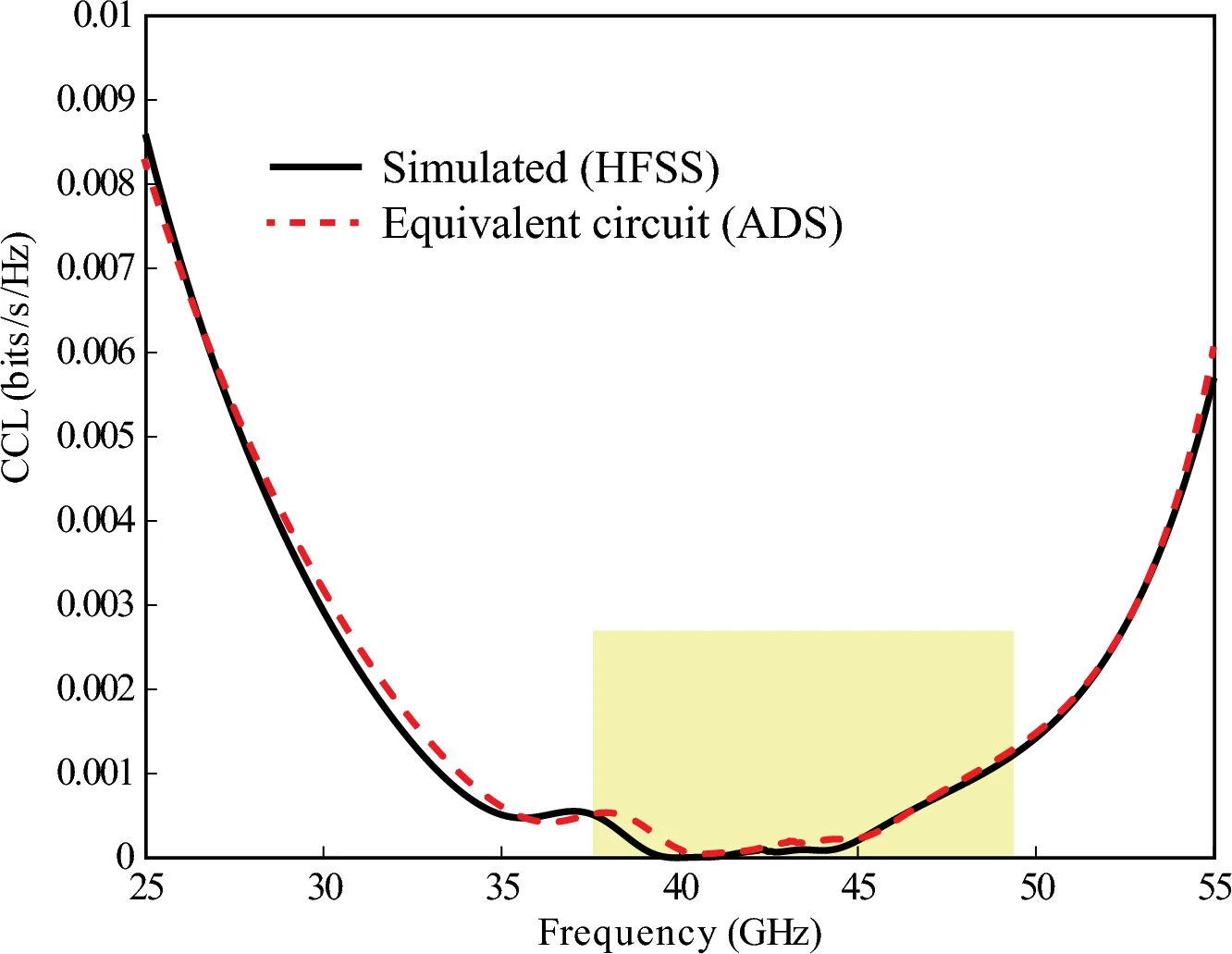
CCL curves of the suggested antenna system.

Channel capacity loss (CCL) in all ports of the suggested antenna is calculated using equations of the parameters 9, 10, 11, and 12 as reported in^31^. Channel capacity loss of the MIMO antenna refers to loss in data transmission capacity due to poor isolation and mutual coupling if multiple ports transmit data simultaneously. According to work reported in^31^, accepted limit of CCL for efficient transmission and reception of MIMO system is 0.4 bits/s/Hz. The CCL curves through HFSS and ADS of the proposed antenna is illustrated in Fig. 17 and a CCL of less than 0.001 bits/s/Hz is achieved for the entire operating band. Based on the study in this section, it is concluded that the proposed antenna offers excellent MIMO characteristics at mmWave band, thereby it is most suitable component for next generation wireless communication.

9 $$\begin{aligned} \textrm{CCL} = -\log _{2}\left( \det (\rho )\right) \end{aligned}$$

10 $$\begin{aligned} \begin{aligned} \rho = \begin{bmatrix} \rho _{11} & \rho _{12} & \rho _{13} & \cdots & \rho _{1,16} \\ \rho _{21} & \rho _{22} & \rho _{23} & \cdots & \rho _{2,16} \\ \rho _{31} & \rho _{32} & \rho _{33} & \cdots & \rho _{3,16} \\ \vdots & \vdots & \vdots & \ddots & \vdots \\ \rho _{16,1} & \rho _{16,2} & \rho _{16,3} & \cdots & \rho _{16,16} \end{bmatrix} \end{aligned} \end{aligned}$$

11 $$\begin{aligned} \rho _{ii} = 1 - \sum _{n=1}^{16} \left| S_{i,n} \right| ^2 \end{aligned}$$

12 $$\begin{aligned} \rho _{ik} = -\left| \sum _{j=1}^{16} S_{ij}^{*} S_{jk} \right| , \quad 1 \le i \le 16,\; 1 \le k \le 16 \end{aligned}$$

**Fig. 18:**
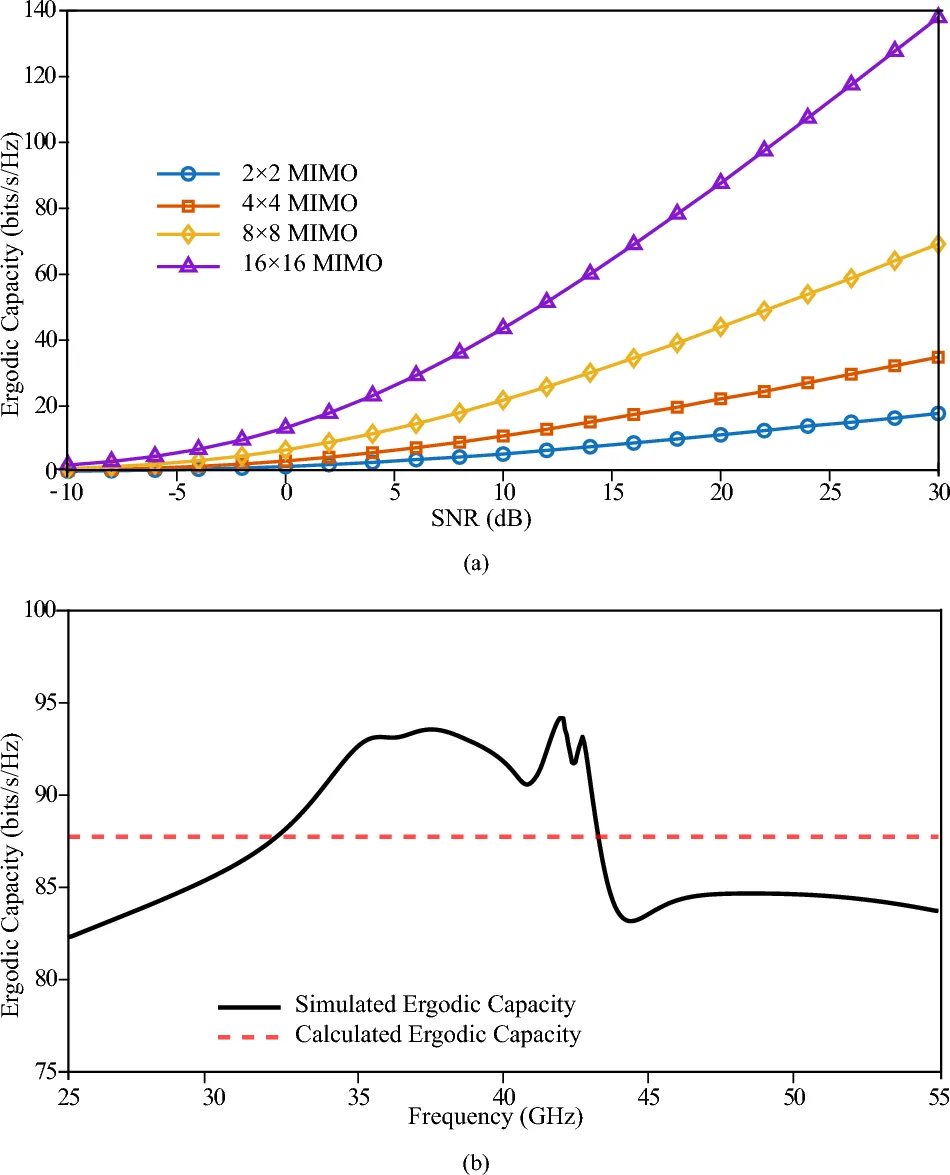
(**a**) Ergodic channel capacity of different MIMO system (**b**) Simulated and ideal Ergodic channel capacity.

The maximum achievable data rate in fading environment of the proposed 16-port MIMO antenna is illustrated in Fig. 18. The ergodic channel capacity for *2 × 2*, *4 × 4*, *8 × 8* and *16 × 16* MIMO antennas with different signal to noise ratio (SNR) is presented in Fig. 18a while the simulated and calculated ergodic channel capacity at the 20 dB SNR value is illustrated in Fig. 18b. From Fig. 18a, it can be concluded that the data rate of the proposed MIMO system increases with the number of MIMO elements. For example, at 20 dB SNR, the ideal capacities for *2 × 2*, *4 × 4*, *8 × 8* and *16 × 16* MIMO systems are observed to be 11.5 $\mathrm {bit/s/Hz}$, 23 $\mathrm {bit/s/Hz}$, $\mathrm {bit/s/Hz}$ and 87 $\mathrm {bit/s/Hz}$, respectively. The ideal Ergodic channel capacities were calculated using Eq. 13 in MATLAB assuming ideal conditions^32^. Subsequently, simulated and calculated channel capacity curves at 20 dB SNR in terms of frequency of the proposed 16-port MIMO antenna are drawn by using simulated s-parameter results and expression as given in Eq. 13. In Eq. 13, channel matrix $H = N_{T} \times N_{R}$ is calculated from s-parameters (real and imaginary), $N_{T}$ - number of transmitting antennas, $N_{R}$ is number of receiving antennas, I - identity matrix, $H^H$- conjugate transpose of channel matrix H. Figure 18b shows that the channel capacity of the proposed antenna varies in between 83-94 bit/s/Hz at 20 dB SNR which is closer to the ideal value of *16 × 16* MIMO antenna system.

13 $$\begin{aligned} CC=\left\{ \log _2{\left[ \det {\left( I+\frac{SNR}{N_T}HH^H\right) }\right] }\right\} \end{aligned}$$

Therefore, the study shows that the proposed antenna is an efficient antenna element for a high data rate communication system.

**Fig. 19:**
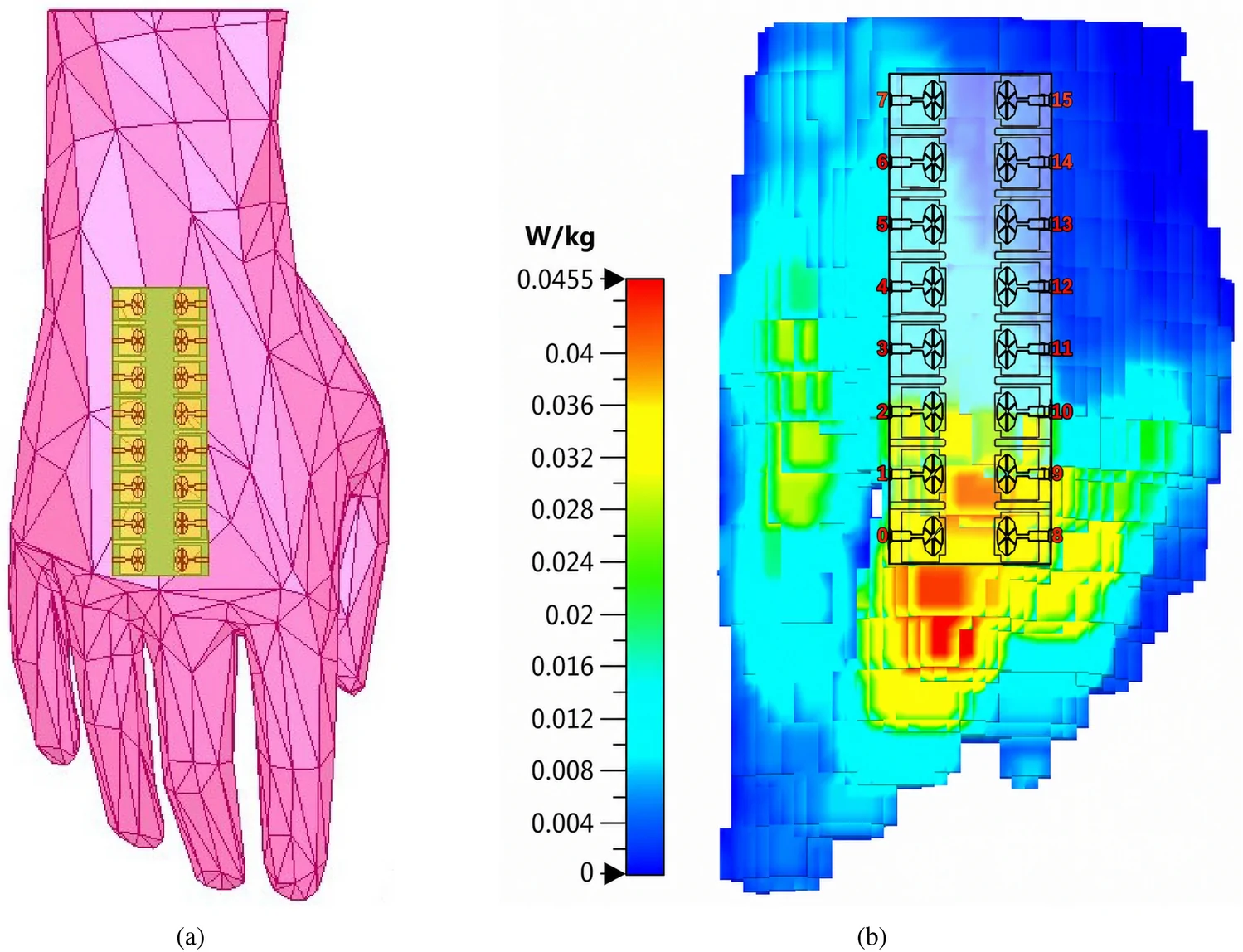
SAR of the proposed antenna at 40 GHz (**a**) Human right-hand phantom model (**b**) EM wave radiation on the human body.

## Specific absorption rate (SAR)

The specific absorption rate (SAR) analysis of the proposed antenna is conducted by placing the antenna on the right hand of a human male model. The SAR value of a mobile phone specifically measures the rate at which electromagnetic waves (EM) are absorbed by the human body when it is in direct contact with the device. A deeper penetration of EM waves into human body tissues results in a higher SAR value, which may be hazardous to human health. As per the guidelines of the Federal Communications Commission (FCC) and the International Commission on Non-Ionizing Radiation Protection (ICNIRP), the safety limit for SAR is 1.6 W/kg averaged over 1 gram of human tissue for mobile devices. The SAR value is a function of conductivity, mass density, and electric field, as given in Eq. 14.

14 $$\begin{aligned} \textrm{SAR} = \frac{\text {Radiated power dissipated in tissue}}{\text {Tissue mass}} = \frac{Conductivity \left| Electric\ field \right| ^2}{Mass\ density} \end{aligned}$$

The proposed antenna is tested on a human hand model for RF exposure at 40 GHz resonating frequency, as shown in Fig. 19a, b. Fig. 19a illustrates the human right-hand model that interacts with the 16-port antenna, while Fig. 19b presents the SAR analysis corresponding to Fig. 19a. All ports of the proposed antenna are simultaneously excited with an input power of 1 W, of which 0.966 W power is accepted, and 0.0045 W power is absorbed by the human tissue. From Fig. 19b, a maximum SAR value of 0.045 W/kg is observed, which is within the safety limits. This low SAR value (0.045 W/kg) makes the proposed antenna suitable for mobile applications at mmWave.

## Comparative study

In this section, a performance study of multi-port MIMO antennas for mmWave applications is conducted. The performance study of the antennas^17,18^^33–38^ as enumerated in Table 1 includes antenna size, operating band, isolation, gain, ECC, CCL and inter-element distance between two elements of the MIMO antenna. According to a literature survey and to the best knowledge of the authors, 16-port MIMO antennas for mmWave applications are rarely reported in the literature. Hence, available antennas with 16-ports^17,18,33^-^35^ and other multi-port antennas such as 4-port^36^, 8-port^37^, and 10-port^38^ antennas have been taken for performance comparison.

The reported antennas such as^38^ have been demonstrated to be highly gainful and of compact geometry, but were restricted to a very limited mmWave range of about 60 GHz, making them less useful in wideband or multi-service applications. The antenna designs^17^ and^33^ showed good isolation values of up to 25 dB and 54 dB respectively, and good gain performance, but^17^ occupies an extremely long structure, while^33^ has a very high envelope correlation coefficient (ECC), and thus poor diversity performance. The antenna^34^, offers a moderate isolation level, but the gain (8.7 dB) is much lower, and that could influence the reliability of the links in real 5G/mmWave applications. The setup suggested in^18^ allows dual-band operation, which is better than frequency agility, but has no reported diversity metrics, including ECC and channel capacity loss (CCL) and therefore cannot be fully analyzed. On the same note,^36,37^, and^38^ offer smaller geometries and high ECC; however, one of them has fewer ports or has relatively lower gain and isolation, restricting their application in high-capacity massive MIMO systems (Table 1).

**Table 1:** Performance study of multi-port MIMO antenna for mmWave applications.

Ref.	Year	Size (mm$^2$)	Band (GHz)	ISO (dB)	Gain (dB)	ECC	CCL	D (mm)	Ports
^17^	2024	30*×*200	25.5–29	25	19.9	0.008	0.4	6.8	16
^33^	2025	43.5*×*43.5	20.6–34.2	54	24.4	0.26	NR	9	16
^34^	2019	48.5*×*21.8	59.1–60.6	27	19.8	NR	NR	NR	16
^35^	2025	60*×*60	28–29.5	35	8.7	NR	NR	NR	16
^18^	2025	52*×*52	30–33 33.5–40.9	22	NR	NR	NR	6	16
^36^	2023	36.2*×*36.2	37.2–39.2	25	10	0.005	0.4	>6	4
^37^	2024	42*×*18	28.5–35.3	17	6	0.001	NR	5	8
^38^	2022	100*×*16	24.25–27.5	35	5.3	0.003	0.005	>5	10
This work	–	26.2*×*79	37.5–49	20	17.9	0.003	0.001	4	16

* NR-* Not reported,* ISO-* Isolation, *D-* Distance between two elements

The proposed antenna provides a balanced and optimized trade-off between compact size, large operating bandwidth, and increased diversity features. In contrast to most of the reported designs, it operates over wideband in 37.5–49 GHz and has a high gain of 17.9 dB and good isolation of 20 dB at all 16-ports. It also has very low values of ECC and CCL indicating a high level of diversity and low mutual coupling effects of the antenna. In addition, the design has relatively short distances between elements, but it also has sixteen ports, which is difficult to achieve in mmWave massive MIMO designs. This wide bandwidth, large number of ports, excellent radiation performance and excellent diversity metrics and features define the originality and practical applicability of the proposed antenna in next-generation high-capacity mmWave wireless communication networks.

## Conclusion

A compact (26.2 *×* 79 *×* 0.508 mm$^3$) 16-port parasitically massive MIMO antenna for mmWave application has been presented and extensively studied. Despite consisting of 16-elements, the proposed antenna exhibits a wide range (37.5–49 GHz) of operational frequency to cover three important mmWave spectrum; n259 (39.5–43.5 GHz), n260 (37–40 GHz) and n262 (47.2–48.2 GHz). The large bandwidth of the proposed antenna is an outcome of the parasitically coupled driven element and impedance matching network, wherein secondary resonance introduced by parasitic elements reduces quality factor and thereby significant improvement in antenna bandwidth. A high gain (17.9 dB) and isolation (> 20 dB) of the intended antenna shows that the antenna maintains stable radiation patterns and strong mutual coupling between the antenna elements. In addition, the antenna shows an excellent value for each performance metric (ECC = 0.003, DG *≈* 10 dB, MEG < −3 dB, TARC < −10 dB, CCL = 0.001 bits/s/Hz). The simulated results have been successfully validated with equivalent circuit results. However, a marginal deviation has been observed between HFSS and ADS results due to different algorithms of the simulating tools, assumptions involved in the equivalent circuit, and high frequency (mmWave) operation. Overall, owing to the antenna performance, the proposed antenna establishes a good degree of balance between all the performance metrics. Interestingly, the antenna offers outstanding features with a very small inter element distance (4 mm) between the antenna elements which makes the antenna suitable for next generation communication technology.

## Data Availability

Datasets were not generated or analyzed during the current study.
